# Targeting primary cilia – associated signaling in glioblastoma: guided approach for drug development

**DOI:** 10.18632/oncoscience.475

**Published:** 2019-01-31

**Authors:** Yuriy Loskutov, Elena N. Pugacheva

**Affiliations:** Department of Biochemistry and West Virginia University Cancer Institute, School of Medicine, Morgantown, WV, 26506, USA

**Keywords:** cilia, glioblastoma, signaling, proliferation, disassembly

Despite unquestionable progress of the recent decades in cancer therapy and anti-cancer drug discovery, the options for glioblastoma (GBM) patients are very limited. Current standard of care includes aggressive surgical resection, radiation therapy, and chemotherapy, however, majority of patients experience recurrence within 32-36 weeks, and median survival is clenching around 10 months. Clearly, there is a need for new strategies to battle GBM. Unfortunately, with GBM we are not on the solid grounds: there is no known common driver mutation to target; the cell lines, commonly used for GBM research, are obsolete, and their origins are in question; even cells GBM arise from, is a topic of harsh debates. Thus, some guidance system would be highly appreciated, a clue towards which signaling cascades are altered and can be targeted.

The primary cilium is a ubiquitous, microtubule-based organelle, an important negative regulator of proliferation and a key sensory organelle. Interestingly, in multiple cancer types, cilia tend to be lost, however, the role of this event is not clear. Modeling the loss of cilia in normal cells suggests that it promotes hyperproliferation in Sonic hedgehog (Shh)-independent cells. In the case of Shh-dependent cell populations such as neural stem cells, ablation of primary cilia seems to inhibit their proliferation and lead to hypocellularity [1]. GBM was reported to have a drastically decreased ciliation rate. Only about 10% of GBM cells assemble cilia. Moreover, electron microscopy analysis of GBM biopsies showed that the few cilia, that they do form, possess multiple structural abnormalities [2]. Further inhibition of primary cilia assembly in patient-derived GBM primary cell culture through the introduction of a dominant-negative variant of KIF3A seems to have a minimal and non-coherent effect on both cell proliferation *in vitro* and tumor growth in the mouse intracranial model [3]. On the other hand, even minimal restoration of primary cilia seems to have a tremendous effect on cell proliferation. This clearly suggests that loss of cilia in GBM is a non-random event, and may be associated with the high proliferative capacity of the tumor cells [4]. It also suggests that GBM are developing either from Shh-independent precursor cells or loose Shh-dependency sometime along with the malignant transformation.

Thus, our group focused on investigating the impact of primary cilia loss in astrocytes, speculated to be one of the potential GBM precursor [5]. In concordance with previous observations in other cell types, loss of primary cilia promotes proliferation of astrocytes, however it was not sufficient to cause malignant transformation. Interestingly, general growth factor response, measured by ERK1/2 and AKT activation, was significantly changed upon loss of primary cilia. We traced it down to aberrant lysophosphatidic acid (LPA) signaling: the translocation of lysophosphatidic acid receptor 1 (LPAR1) to the plasma membrane in the absence of primary cilia promotes binding the alternative G-protein secondary messengers [6], changing the cellular response to the LPA stimulation. In GBM inhibition of LPA signaling was sufficient to decrease proliferation both *in vivo* and *in vitro*, arguing that it is a viable therapeutical approach, which patients might benefit from [7].

LPAR1 is not a unique receptor targeted to primary cilia. A comprehensive list of receptor tyrosine kinases, including PDGFR, EGFR and FGFR, were reported to localize to the cilia [6]. Furthermore, G-protein coupled receptors are very common to be targeted to the cilia, there are at least two independent ciliary targeting sequences within the superfamily [8]. It is clear, that loss of the cilia will effect signaling of at least part of the cascades, in which receptors localize to the cilia. So far, two possible scenarios are confirmed: degradation of the ciliary receptor upon cilia loss (PDGFRαα [6]), and redistribution of the receptor to the plasma membrane (LPAR1 [7]). The systematic studies of the signaling in presence and absence of the cilia will help us to better understand the reasons for decreased ciliation in GBM and other cancer types. At the end, these findings will help us to devise novel targets for anti-cancer therapy. Thus, primary cilium could be the guide pointing us towards successful treatment of GBM.
